# SERS as a Probe of Surface Chemistry Enabled by Surface-Accessible Plasmonic Nanomaterials

**DOI:** 10.1021/acs.accounts.3c00207

**Published:** 2023-07-12

**Authors:** Yikai Xu, Yingrui Zhang, Chunchun Li, Ziwei Ye, Steven E. J. Bell

**Affiliations:** † Key Laboratory for Advanced Materials and Feringa Nobel Prize Scientist Joint Research Center, Frontiers Science Center for Materiobiology and Dynamic Chemistry, School of Chemistry and Molecular Engineering, East China University of Science and Technology, 130 Meilong Road, Shanghai 200237, P. R. China; ‡ School of Chemistry and Chemical Engineering, Queen’s University Belfast, University Road, Belfast BT7 1NN, United Kingdom; ∥ Key Laboratory for Advanced Materials, Joint International Research Laboratory of Precision Chemistry and Molecular Engineering, Feringa Nobel Prize Scientist Joint Research Center, School of Chemistry and Molecular Engineering, East China University of Science and Technology, Shanghai 200237, P. R. China

## Abstract

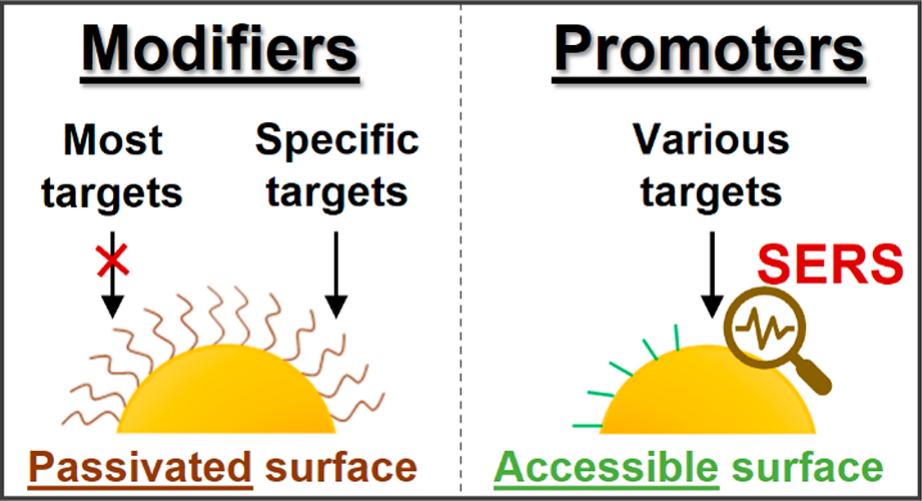

When the size of materials is reduced, their volume decreases much faster than their surface area, which in the most extreme case leads to 2D nanomaterials which are “all surface”. Since atoms at the surface have free energies, electronic states, and mobility which are very different from bulk atoms, nanomaterials that have large surface-to-volume ratios can display remarkable new properties compared to their bulk counterparts. More generally, the surface is where nanomaterials interact with their environment, which in turn places surface chemistry at the heart of catalysis, nanotechnology, and sensing applications. Understanding and utilizing nanosurfaces are not possible without appropriate spectroscopic and microscopic characterization techniques. An emerging technique in this area is surface-enhanced Raman spectroscopy (SERS), which utilizes the interaction between plasmonic nanoparticles and light to enhance the Raman signals of molecules near the nanoparticles’ surfaces. SERS has the great advantage that it can provide detailed *in situ* information on surface orientation and binding between molecules and the nanosurface. A long-standing dilemma that has limited the applications of SERS in surface chemistry studies is the choice between surface-accessibility and plasmonic activity. More specifically, the synthesis of metal nanomaterials with strong plasmonic and SERS-enhancing properties typically involves the use of strongly adsorbing modifier molecules, but these modifiers also passivate the surface of the product material, which prevents the general application of SERS in the analysis of weaker molecule–metal interactions.

In this Account, we discuss our efforts in the development of modifier-free synthetic approaches to synthesize surface-accessible, plasmonic nanomaterials for SERS. We start by discussing the definition of “modifiers” and “surface-accessibility”, especially in the context of surface chemistry studies in SERS. As a general rule of thumb, the chemical ligands on surface-accessible nanomaterials should be easily displaceable by a wide range of target molecules relevant to potential applications. We then introduce modifier-free approaches for the bottom-up synthesis of colloidal nanoparticles, which are the basic building blocks for nanotechnology. Following this, we introduce modifier-free interfacial self-assembly approaches developed by our group that allow the creation of multidimensional plasmonic nanoparticle arrays from different types of nanoparticle-building blocks. These multidimensional arrays can be further combined with different types of functional materials to form surface-accessible multifunctional hybrid plasmonic materials. Finally, we demonstrate applications for surface-accessible nanomaterials as plasmonic substrates for SERS studies of surface chemistry. Importantly, our studies revealed that the removal of modifiers led to not only significantly enhanced properties but also the observation of new surface chemistry phenomena that had been previously overlooked or misunderstood in the literature. Realizing the current limitations of modifier-based approaches provides new perspectives in manipulating molecule–metal interactions in nanotechnology and can have significant implications in the design and synthesis of the next generation of nanomaterials.

## Key References

- Ye, Z.; Li, C.; Celentano, M.; Lindley, M.; O’Reilly, T.; Greer, A. J.; Huang, Y.; Hardacre, C.; Haigh, S. J.; Xu, Y.; Bell, S. E. J. Surfactant-Free Synthesis of Spiky Hollow Ag–Au Nanostars with Chemically Exposed Surfaces for Enhanced Catalysis and Single-Particle SERS. *JACS Au***2022**, *2*, 178–187.^1^ A simple and versatile modifier-free synthetic approach using Ag^+^ as the growth-directing agent for the synthesis of plasmonic bimetallic colloidal nanoparticles with accessible surfaces. This paper also systematically demonstrates the enhanced functionalities that come with creating accessible surfaces.
- Xu, Y.; Konrad, M. P.; Lee, W. W. Y.; Ye, Z.; Bell, S. E. J. A Method for Promoting Assembly of Metallic and Nonmetallic Nanoparticles into Interfacial Monolayer Films. *Nano Lett.***2016**, *16*, 5255–5260.^2^ A general and modifier-free approach for assembling charged colloidal nanoparticles at water–oil interfaces to form 2-dimensional nanoparticle arrays.
- Zhang, Y.; Ye, Z.; Li, C.; Chen, Q.; Aljuhani, W.; Huang, Y.; Xu, X.; Wu, C.; Bell, S. E. J.; Xu, Y. A General Approach for Constructing Surface-Accessible Emulsions to Unlock Plasmonic Sensing and Catalytic Applications. *Nat. Commun.***2023**, *14*, 1392.^3^ A general and modifier-free approach for the creation of stable Pickering emulsions from charged colloidal nanoparticles with enhanced functionalities in SERS and interfacial catalysis.
- Li, C.; Chen, Z.; Huang, Y.; Zhang, Y.; Li, X.; Ye, Z.; Xu, X.; Bell, S. E. J.; Xu, Y. Uncovering Strong π-Metal Interactions on Ag and Au Nanosurfaces Under Ambient Conditions via *in situ* Surface-Enhanced Raman Spectroscopy. *Chem***2022**, *8*, 2514–2528.^4^ Unraveling the existence of π-metal interactions on Ag and Au nanosurfaces under ambient conditions using SERS and surface-accessible interfacial nanoparticle films. This interaction was not realized previously due to the extensive use of modifiers in the construction of SERS substrates.

## 1 Introduction

Plasmonic nanomaterials, in particular Ag and Au, have important applications in a wide range of areas including energy production, biosensing, photodynamic therapy, etc.^5,6^ Regardless of the exact application, the interactions between molecules and the metal nanosurfaces always play a central role in achieving the desired functions. This places surface chemistry at the heart of nanoscience.

Currently, a multitude of characterization techniques are available for the analysis of the surface chemistry of nanomaterials. For example, X-ray photoelectron spectroscopy (XPS) provides large amounts of chemical information that allows molecule–metal interactions to be probed at an atomic level.^7^ However, XPS studies are typically run under ultrahigh vacuum conditions, while the majority of applications take place in solution or in gaseous environments. Scanning-tunneling microscopy and nanoIR spectroscopy can be used to obtain rich chemical information under ambient conditions while allowing the presence of some specific solvents,^8,9^ but this is still far from the complex environments that are relevant to most applications. It is possible to perform *in situ* analysis using techniques, such as FTIR spectroscopy and cyclic voltammetry;^10,11^ however, the chemical information provided by these techniques is typically limited by low sensitivity and chemical specificity. Therefore, it is vital to develop sensitive characterization techniques that allow the surface chemistry of nanomaterials to be probed in the complex environments that are directly relevant to their applications.

Surface-enhanced Raman spectroscopy (SERS) is an analytical technique which potentially satisfies the simultaneous requirement for sensitivity, molecular specificity and sampling versatility needed for surface chemistry analysis of nanomaterials in complex environments.^12−14^ In general, SERS is achieved by placing the analyte molecules near/on the surface of plasmonic materials (often referred to as the “enhancing substrates”) which are able to enhance their Raman signals by hundreds to millions of times. With the appropriate enhancing substrate, SERS can be performed *in situ* to probe molecule–metal interactions down to a single-molecule level, with specific information on the surface orientation and formation/breaking of chemical bonds.^15,16^ The key to applying SERS to surface chemistry studies in complex environments is to avoid blocking access to the metal surface since this will prevent the desired molecule–metal interactions.^17−19^ This is a major challenge, since the synthesis of metal nanomaterials with strong plasmonic properties relies heavily on the use of molecular modifiers, which adsorb strongly to the nanosurface to direct crystal growth, provide stability and induce self-assembly.^20,21^

**Figure 1:**
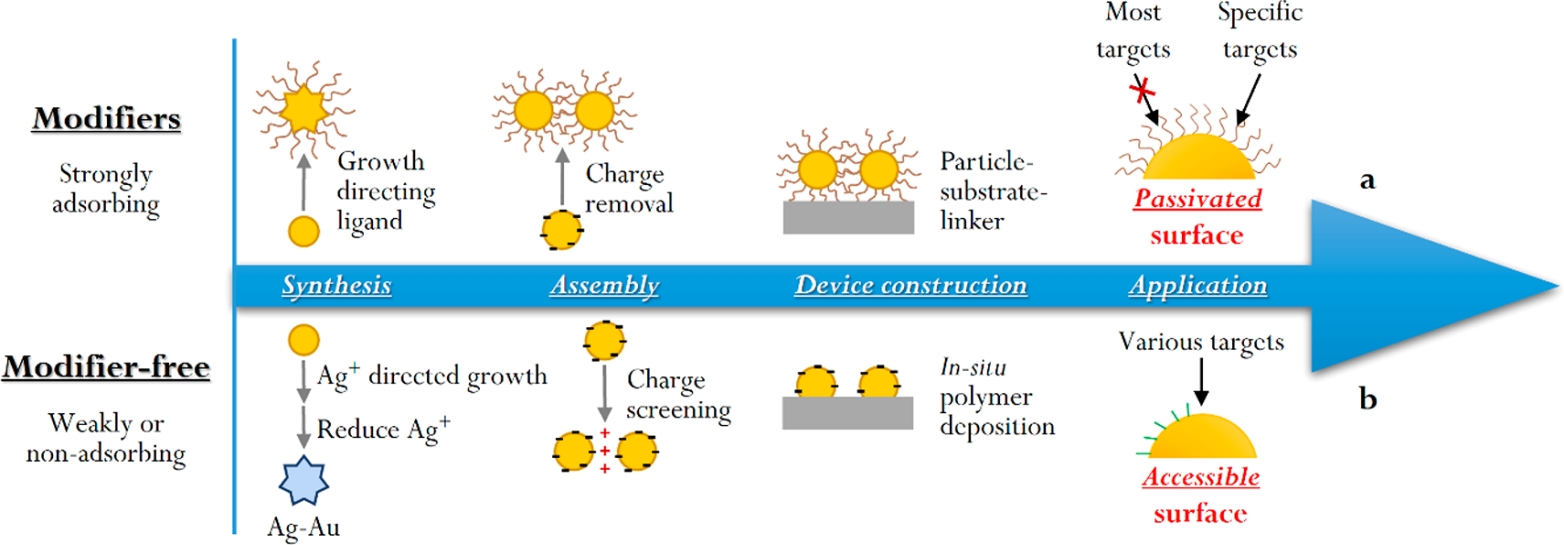
Illustrations comparing the construction and applications of plasmonic nanomaterials with/without modifiers.

Here we describe our recent progress in the development of modifier-free approaches for the synthesis and stabilization,^1^ assembly^2,3,22−24^ and device construction^25−30^ of surface-accessible plasmonic nanomaterials (Figure 1). The ability to synthesize highly active engineered plasmonic substrates that retain their surface-accessibility, even in complex environments, paves the way for using SERS to study chemical phenomena at molecule–metal interfaces in a way that is not possible with traditional modifier-capped plasmonic substrates. This has allowed us to develop new insights into fundamental surface chemistry^4,31−36^ and enabled the design of significantly improved plasmonic sensors for important real-life analytes including pharmaceuticals, explosives, “legal highs”, DNA, etc.^1,3,37−42^ More generally, we have shown that the understanding of exposed surfaces which SERS provides can underpin the development of nanomaterials with enhanced and/or novel functionalities that are significant for applications beyond sensing, for example, in catalysis^1,3,25,26,43^ and electronics.^25^

## 2 Modified or Accessible Nanosurfaces

Currently, although strongly adsorbing chemical ligands are extensively used in the construction of nanomaterials, there is no widely accepted definition of “modifiers” or “surface-accessible nanomaterials”. Therefore, for clarity, Figure 1a shows examples of the use of modifiers, which we define as chemical ligands that are adsorbed strongly on the surface of the product for various purposes and which affect the functionality of the material. Conversely, Figure 1b illustrates modifier-free approaches for the creation of surface-accessible nanomaterials. It is important to differentiate the surface-accessibility discussed in this Account from the clean surfaces that have been traditionally used in fundamental studies performed under ideal conditions in ultrahigh vacuum.^44^ In the current case, the nanomaterials studied by SERS are placed under ambient conditions that represent the chemical environment in which the majority of their applications are carried out. The surface of the nanomaterials is undoubtedly covered by chemical species ranging from solvent molecules to organic capping ligands, but if these can be easily displaced, the surfaces of the materials will still be accessible.

It is now well-established that the adsorption strength of ligands on nanosurfaces is governed by a wide range of factors, including the chemical structure of the ligand, the crystal structure of the nanosurface and the chemical environment in which adsorption takes place.^45,46^ Indeed, we have recently shown that the binding energy of Cl^–^ onto Au nanoparticle surfaces drops from −0.77 to −0.13 eV with increasing surface coverage.^4^ Therefore, the role of a particular type of surface-adsorbed molecule, i.e., whether it should be termed a modifier, should be carefully assessed according to the specific conditions. However, a general rule of thumb in the construction of surface-accessible nanomaterials is that the chemical ligands used during material synthesis should be easily displaceable by a wide range of target molecules relevant to their potential applications. This Account is focused on discussing modifier-free synthesis for the creation of plasmonically active surface-accessible Ag and Au nanomaterials for SERS. The main types of surface-accessible materials discussed in this Account are summarized in Table 1. For examples of other types of SERS active surface-accessible nanomaterials, the readers can refer to refs (47−49). Since the majority of target molecules bind to Ag and Au nanomaterials through sulfur or nitrogen functional groups,^50^ this means that, generally speaking, ligands which bind more weakly to Ag and Au than thiols, disulfides, pyridines, etc. are more likely to be suitable for creating surface-accessible nanomaterials in SERS. More broadly speaking, since a wide range of nanotechnologies rely on surface interactions, the concept of modifiers and surface-accessibility is significant to a variety of applications far beyond SERS. A typical example of this is in catalysis, where it has been shown that strongly adsorbed bulky polymeric ligands, such as polyvinylpyrrolidone (PVP), can significantly decrease catalytic activity.^51^

**Table 1:** Summary of Surface-Accessible Ag and Au Nanomaterials and Their Applications as Enhancing Substrates for SERS Studies of Surface Chemistry

Material type	Example material	Capping ligand	Synthetic approach	Question addressed	Ref
Colloid	Spiky and hollow Ag–Au nanostars	Cl^–^	Ag^+^-directed growth in wet synthesis	Effect of capping ligands on niraparib adsorption	(1)
Assembly	2-Dimensional interfacial nanoparticle films	Cl^–^, citrate ion	Promoter-assisted self-assembly	Uncovering π-metal interactions under ambient conditions	(4)
	Pickering emulsion	Cl^–^, citrate ion	Promoter and stabilizer assisted self-assembly	Interference on adenine adsorption by modifiers	(3)
	Colloidosome	Citrate ion	Ultrasonication combined with promoter assisted self-assembly	Probing adenine adsorption kinetics in artificial serum	(24)
	Agglomerated colloid	Cl^–^, BH_4_^–^, citrate ion	Salt-induced agglomeration	Label-free detection of DNA/RNA	(37)
Composite	Deposited 2-dimensional nanoparticle films	Cl^–^, citrate ion	Dip-coating	Monitoring disulfide adsorption from bacteria headspace	(40)
	Nanoparticle-decorated polymer composites	Citrate ion	*In situ* polymer deposition	Probing solvent-free molecule–metal interactions	(41)
	Polymer encapsulated agglomerates	Cl^–^, citrate ion	Mixing and drying	Using drug–Ag interactions for sensing	(27)

## 3 Surface-Accessible Colloids

The first step to constructing surface-accessible nanomaterials is to synthesize modifier-free nanoparticles since they are the basic building blocks in nanotechnology. This can be achieved through methods such as laser ablation^47^ and physical vapor deposition,^43^ but we have focused our efforts on developing modifier-free colloidal synthetic procedures since this offers a good balance between morphological control, product yield and cost and is potentially more efficient than postprocessing materials to remove modifiers.^19,49^ The simplest method for forming Ag and Au colloids is to react a metal precursor and reducing agent to create metal atoms which nucleate and grow into larger nanoparticles.^52^ This mechanism underpinned the development of the earliest types of colloidal Ag and Au nanoparticles including citrate-reduced Ag and Au colloids,^53,54^ hydroxylamine-reduced Ag colloid^55^ and sodium borohydride-reduced Ag colloid,^56^ which were modifier-free and provided good SERS enhancement when agglomerated to form interparticle plasmonic hot spots.^31−35,37−39^ However, these colloids consisted of nanoparticles that had poorly controlled structures and broad size distributions. The traditional approach to obtain nanoparticles with well-controlled morphologies is to use molecular modifiers that adsorb to particular facets of the nanocrystal to guide its growth. While highly effective, this approach inevitability leads to the product particle being covered by strongly adsorbed modifiers. Therefore, it is important to develop new types of growth-directing agents that provide morphological control without passivating the product surface.

**Figure 2:**
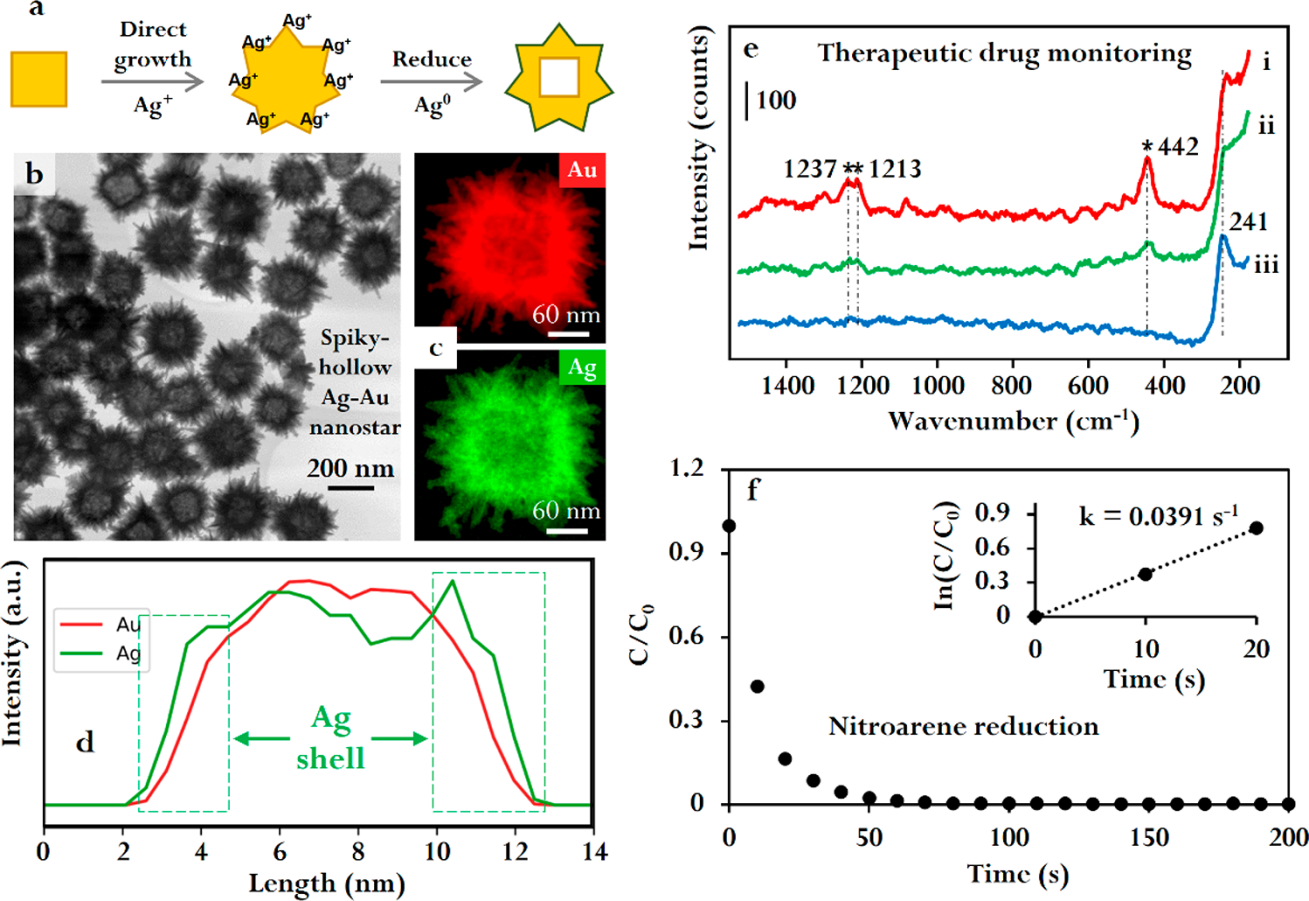
(a) Schematic illustration of the formation of surface-accessible nanostars. (b) Scanning transmission electron microscopy (TEM)–high-angle annular dark field images of spiky hollow nanostars. (c, d) Energy-dispersive X-ray elemental mapping of a nanostar. (e) Surface-enhanced Raman spectroscopy (SERS) spectra of 10^–5^ M doxorubicin adsorbed on (i) pristine nanostar colloid and (ii) polyvinylpyrrolidone-stabilized nanostar colloid. Spectrum (iii) shows the blank SERS signals from the pristine nanostars. (f) Plots showing the decrease in the concentration of 4-nitrophenol with time in the NaBH_4_ reduction of 4-nitrophenol catalyzed by nanostars. Adapted with permission from ref (1). Copyright 2021 American Chemical Society.

It has been shown that Ag^+^ can adsorb selectively to the (100) plane of Au nanocrystals, which has allowed Ag^+^ to be used as the growth-directing agent in the synthesis of Au nanoparticles with unique morphologies.^57^ We have developed a simple and versatile modifier-free synthetic approach using Ag^+^ as the growth-directing agent (Figure 2a).^1^ Importantly, unlike conventional modifiers, the Ag^+^ ions are reduced to Ag^0^ and become part of the plasmonic metal nanoparticle after acting as the growth-directing agent, which makes the surface of the product accessible. Based on this method, various new types of surface-accessible colloidal Au nanoparticles could be created in minutes, at room temperature, with near 100% morphological yield. For example, Figures 2b-d show spiky, hollow Ag–Au nanostars formed using this modifier-free synthesis. The SERS spectrum (iii) in Figure 2e shows that the surface of the product particles only contained a small amount of Cl^–^, which was introduced as part of the metal precursor. As a result, the product nanostars exhibited significantly enhanced plasmonic and catalytic properties compared to their modifier-covered counterparts, which highlights the significance of surface-accessibility. Figures 2e–f show examples of using SERS detection for important analytes, in this case anticancer drugs and the catalytic reduction of 4-nitrophenol.

## 4 Surface-Accessible Nano-Assemblies

Since the coupling between closely packed nanoparticles often leads to enhanced plasmonic properties, Ag and Au nanoparticles often need to be assembled into hierarchical nanostructures to achieve optimal enhancement in SERS. The most straightforward approach to generating plasmonic assemblies is by adding salts to electrostatically stabilized plasmonic colloids, such as the surface-accessible citrate-reduced Ag and Au colloids and hydroxylamine-reduced Ag colloid mentioned above, to form agglomerated nanoparticles. This approach was one of the first methods used for generating reliable SERS spectra and has since been widely adopted by the SERS community.^58^ The key to performing SERS with agglomerated colloids is the selection of an appropriate salt as the aggregating agent. This is because the identity of the salt directly impacts the structure and surface-accessibility of the agglomerate; in particular, salts with ions that strongly adsorb to the surface may compete with the target analyte. We have found that this can compromise the limit of detection for weakly adsorbing analytes^31^ or in extreme cases completely prevent detection.^38^ The fact that colloidal aggregation is a dynamic process that cannot be halted or reversed once initiated is a major drawback for the application of surface-accessible agglomerated nanoparticles as SERS substrates. Even though we have shown that highly reproducible SERS spectra can be obtained with agglomerated nanoparticles, provided that the appropriate experimental technique and equipment are used, their poor stability makes them unsuitable for time-dependent SERS studies.

**Figure 3:**
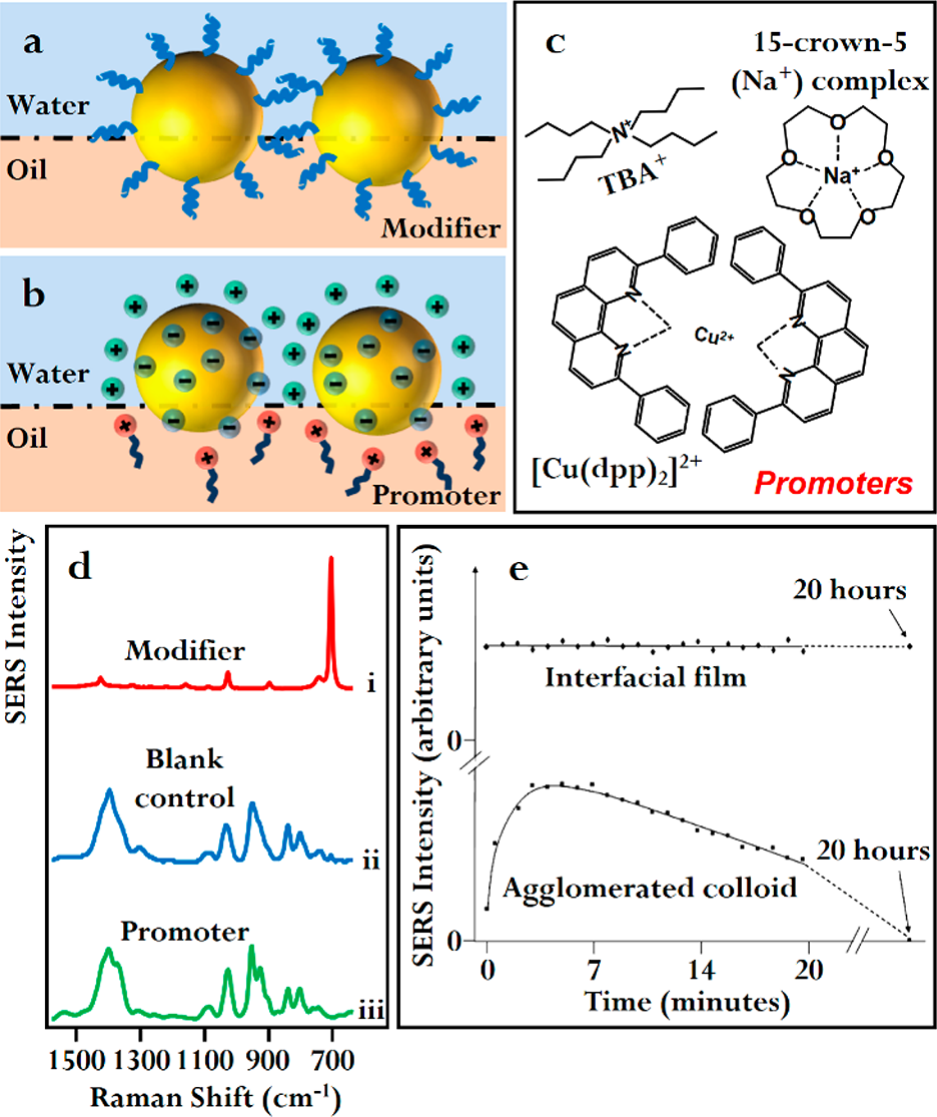
(a, b) Schematic illustration of nanoparticle self-assembly at the interface between water and a denser oil, such as dichloromethane, induced by modifiers and promoters, respectively. (c) Examples of promoters. (d) Typical SERS spectra of (i) an interfacial film formed with modifiers and citrate-reduced Ag nanoparticles, (ii) agglomerated citrate-reduced Ag nanoparticles, and (iii) an interfacial film formed with promoters and citrate-reduced Ag nanoparticles. (e) Plot comparing the SERS signal intensity obtained from agglomerated colloid and interfacial films over time. Adapted with permission from ref (59). Copyright 2021 the authors. Published by Royal Society of Chemistry under a Creative Commons Attribution-NonCommercial 3.0 Unported License. Adapted with permission from ref (22). Copyright 2013 American Chemical Society.

An effective method to generate stable and reproducible SERS-active nanomaterials is to assemble Ag and Au nanoparticles at water–oil interfaces.^59^ In general, the adsorption of solid nanoparticles to the interface of two highly immiscible liquids is an energetically favorable process, which is driven by the reduction in interfacial surface tension. As a result, charge-neutral nanoparticles migrate spontaneously to the water/oil interface to form densely packed films. However, for electrostatically stabilized colloidal nanoparticles, such as the surface-accessible citrate-stabilized Ag and Au nanoparticles, their assembly into closely packed structures is prevented by strong interparticle electrostatic repulsion. Therefore, modifiers are conventionally used to displace the charged capping ligands on Ag and Au nanoparticles, to facilitate the formation of plasmonically active nanoparticle films at water–oil interfaces (Figure 3a).^59^ This approach not only passivates the surface of the nanoparticles but is also highly inconvenient, since the adsorption of modifiers is material-specific, and finding the appropriate modifier relies largely on trial-and-error. To address this problem, we have developed a modifier-free approach to screen the interparticle electrostatic repulsion using organo-electrolytes, which we term “promoters”, that carry an opposite charge to the nanoparticles (Figure 3b).^2,22^Figure 3c shows the chemical structures of several typical types of promoters. Importantly, the promoters do not adsorb onto the nanoparticles but instead dissolve in the oil layer where they reduce interparticle electrostatic repulsion through charge-screening. This leaves the surface chemistry of the nanoparticles unchanged after self-assembly and allows the surface-accessibility of the nanoparticles to be fully retained, as shown by SERS (Figure 3d). Moreover, these interfacial arrays show significantly enhanced SERS stability compared to simple agglomerated colloids (Figure 3e),^22^ which paved the way for important SERS studies of surface chemistry, as discussed later in this Account.

**Figure 4:**
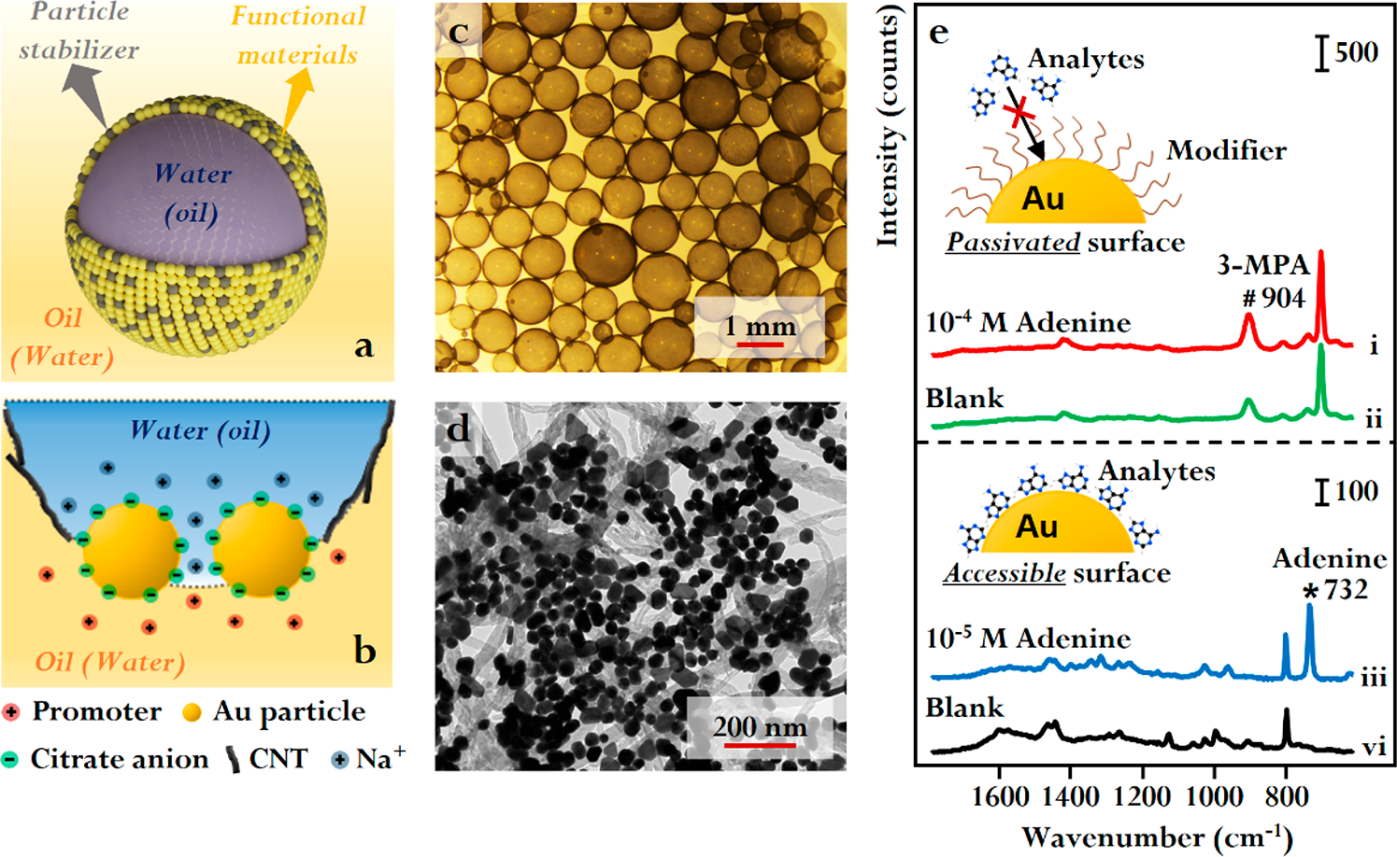
(a) Schematic illustration of a Pickering emulsion droplet with its surface covered in a mixed nanoparticle layer of particle stabilizers and functional materials. (b) Schematic illustration of the effect of promoters and carbon nanotube (CNT) stabilizers in the formation of w/o Pickering emulsions carrying unmodified citrate-reduced Au nanoparticles. (c) Optical microscopy image of a typical sample of CNT-Au with/without Pickering emulsions. (d) TEM image of the CNT and Au nanoparticles on the interface of a CNT-Au w/o emulsion droplet. (e) Comparison of the performance of Au emulsions formed using (i, ii) 3-mercaptopropionic acid (3-MPA) modifiers or (iii, iv) promoters and stabilizers in the SERS detection of adenine. Adapted with permission from ref (3). Copyright 2023 the authors. Published by Springer Nature under a Creative Commons Attribution 4.0 International License.

The promoter-assisted interfacial self-assembly approach can also be readily extended to the construction of plasmonic Pickering emulsions, which have potential as biphasic enhancing substrates for important fundamental SERS studies.^3^ Pickering emulsions consist of fine liquid droplets, which are covered by a layer of solid particles and dispersed in another immiscible fluid. In order to stabilize the emulsion droplets, the nanoparticles must be able to overcome interparticle electrostatic repulsions and, in addition, have a contact angle at the water–oil interface which is within an appropriate range. Since common colloidal Ag and Au nanoparticles do not possess the appropriate surface-wettability to stabilize emulsion droplets, this has meant that the synthesis of plasmonic Pickering emulsions has always required surface modification of the nanoparticles to alter their wettability or remove surface charge. However, this synthetic approach represents a dead-end for SERS applications, since displacing the modifiers from the surface of the nanoparticles with analyte molecules is not only challenging but may also destabilize the emulsion system. We have developed a modifier-free approach for constructing Pickering emulsions with fully customizable nano- or microparticle constituents and whose droplet size, stability, and functional particle coverage can be completely controlled. The key to our approach is the combined use of promoters and stabilizers. The promoters provide charge screening, while the stabilizers are particles which inherently possess the appropriate surface properties to stabilize emulsion droplets, such as carbon nanotubes in the example shown (Figures 4a–b). This method allowed the formation of Pickering emulsions which remained stable for >1 month and also carried a second population of unmodified surface-accessible Ag or Au nanoparticles at the interface (Figures 4c–d). Freeing the emulsions from modifiers unlocks various potential applications that require molecule–surface interactions and cannot be achieved with conventional modifier-capped Pickering emulsions, which, for example, include SERS detection of adenine as well as various other types of weakly-adsorbing analyte molecules (Figure 4e).

**Figure 5:**
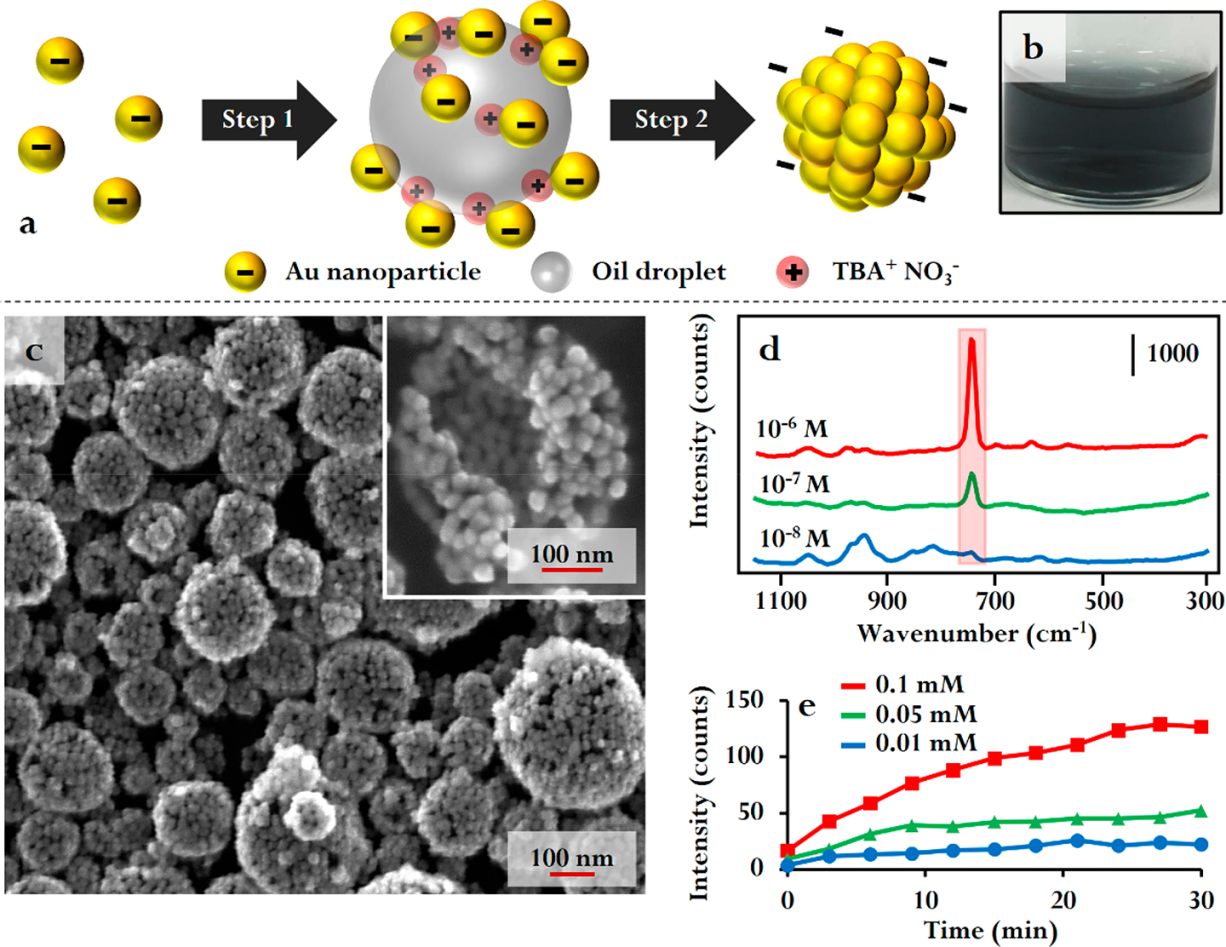
(a) Schematic illustration of the formation of surface-accessible colloidosomes. (b) Optical image of a surface-accessible colloidosome sample formed from citrate-reduced Au nanoparticles. (c) Scanning electron microscopy image of a typical colloidosome sample formed from citrate-reduced Au nanoparticles. (d) SERS signals which were obtained from Ag colloidosomes treated with different concentrations of adenine in artificial serum. (e) Growth of the adenine peak (731 cm^–1^) at various adenine concentrations versus time. Adapted with permission from ref (24). Copyright 2019 Wiley-VCH Verlag GmbH & Co. KGaA, Weinheim.

An alternative approach to utilizing plasmonic emulsions is to convert them to colloidosomes by removing the dispersed phase. This process is typically performed with stable Pickering emulsions whose surfaces are covered in modifiers.^60^ We have shown a modifier-free approach based on ultrasonication and promoter-induced self-assembly that allows the synthesis of stable and plasmonically active surface-accessible colloidosomes from citrate-reduced Ag and Au nanoparticles.^24^ As shown in Figure 5a–b, sonicating an aqueous colloid with a small amount of oil and promoter leads to the formation of o/w Pickering emulsions several hundred nanometers in diameter. The emulsions are not stable since they are covered with unmodified citrate-reduced Ag or Au nanoparticles, but the oil droplets evaporate within minutes before being able to coalesce, which leads to the formation of stable colloidosomes consisting of surface-accessible Ag or Au nanoparticles held together by strong interparticle van der Waals attraction (Figure 5c). Similar to salt-aggregated colloids, these colloidosomes possess 3-dimensional plasmonic hot-spots which make them even more plasmonically active than the parent emulsions. At the same time, since the weakly adsorbing charged ligands remain on the surface of the colloidosomes, the colloidosomes remain as stable dispersions in solution for weeks. Importantly, the preformed plasmonically active interparticle nanogaps in the colloidosomes act as molecular sieves to prevent the adsorption of albumin into the plasmonic hot-spots, which helps combat biofouling. This along with the accessibility of the plasmonic nanosurface paved the way for SERS quantitation and kinetic studies in artificial serum. For example, as shown in Figure 5d–e, the experiments revealed that the adsorption kinetics of adenine in artificial serum varied dramatically depending on its concentration, which is important for developing a unified procedure to achieve reliable quantitative SERS.

## 5 Surface-Accessible Composite Materials

**Figure 6:**
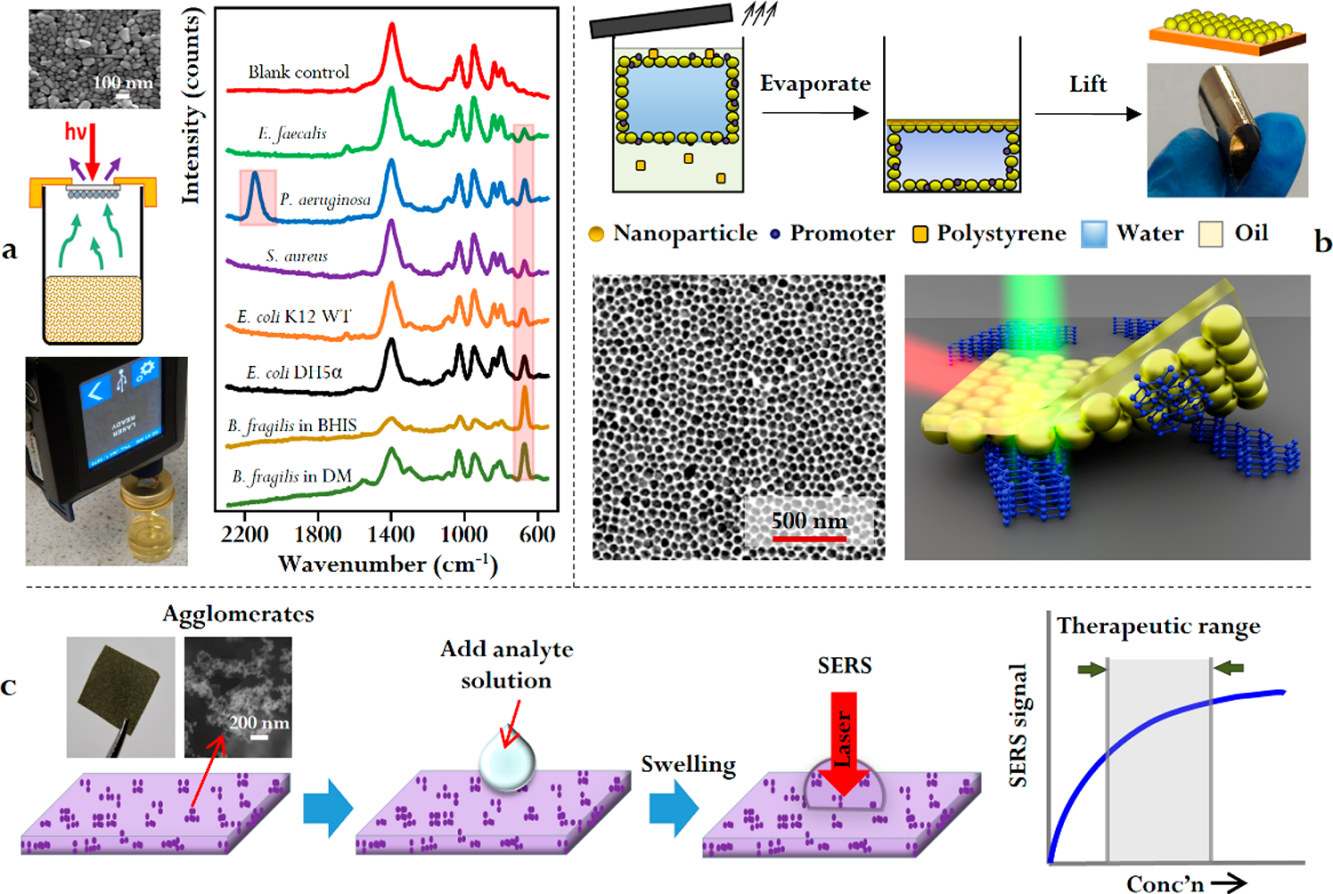
(a) Panel showing the application of 2-dimensional citrate-reduced Ag nanoparticle films deposited on quartz in the SERS analysis of bacteria headspace. Adapted with permission from ref (40). Copyright 2018 Wiley-VCH Verlag GmbH & Co. KGaA, Weinheim. (b) Panel showing the synthesis and application of surface-exposed nanoparticle films as flexible substrates for solvent-free SERS. Adapted with permission from refs (25) and (41). Copyright 2017 Wiley-VCH Verlag GmbH & Co. KGaA, Weinheim, and 2018 Elsevier, Ltd., respectively. (c) Panel showing the synthesis and application of agglomerated colloid-hydroxyethyl cellulose films in SERS therapeutic drug monitoring. Adapted with permission from ref (29). Copyright 2016 Elsevier, Ltd.

The surface-accessible colloidal nanomaterials can be combined with a range of substrate materials to form composite materials with new functionalities and enhanced stability that can benefit SERS applications. The most straightforward way to construct composite materials containing nanoparticles is to physically deposit the colloidal nanomaterials onto solid substrate materials.^61,62^ This method is particularly effective for transferring 2-dimensional Ag and Au interfacial assemblies onto hydrophilic solid materials, such as glass or metal. Since the surface-accessible Ag and Au nanoparticles are typically hydrophilic, they adhere to the surface of hydrophilic materials so they can be transferred onto solid supports via a simple dip-coating process.^22,40^ Importantly, after the dip-coating process, the nanoparticle layer is held to the surface of the substrate material by favorable van der Waals interactions and does not show obvious shedding over time or when dipped repeatedly into solvents.^63^ This is an important feature for performing long-term SERS kinetic studies, for example, we have studied the adsorption kinetics of biomarkers from the headspace of bacterial cultures using 2-dimensional arrays of Ag and Au nanoparticles deposited on quartz (Figure 6a).^40^

Although convenient, the nature of the physical deposition approach means that it is sometimes irreproducible and often disrupts the structure of the nanoassembly. To fully retain the packing order and in turn the plasmonic properties of interfacial assemblies, we have developed an *in situ* polymer deposition approach.^25,26^ As shown in Figure 6b, polymers, such as polystyrene, may be predissolved in the oil phase used for interfacial self-assembly without affecting the migration of the nanoparticles to the interface. After the interfacial nanoparticle films form, the sample is allowed to rest under ambient conditions, so that the oil phase slowly evaporates. This leads to the precipitation of the predissolved polystyrene at the oil side of the interface, which fixes the particles *in situ* and results in the formation of a freestanding polystyrene sheet with the nanoparticles anchored on the surface (a “surface-exposed nanoparticle film”). Since this *in situ* polymer deposition process occurs only at the oil side of the interface, the surface of the particles facing the aqueous solvent remains physically and chemically exposed but, in contrast to the deposited films, the nanoparticle–polymer films are much more robust and can even be scratched without visible particle shedding.^25^ Moreover, the same approach can also be readily used to transform Pickering emulsions into polymer microbeads with a layer of nanoparticles anchored on the surface.^26^ Importantly, the physical robustness and accessible surface chemistry of these nanoparticle-decorated polymer composites means that they can be used as highly versatile platforms for studying molecule–metal interactions in complex sampling environments ranging from crossing ink lines to river water using SERS.^26,41^

A drawback with the composite materials formed by using *in situ* polymer deposition is their relatively poor long-term stability. Since the nanoparticles are directly exposed to air and are therefore prone to surface oxidation and Ostwald ripening, their SERS activities typically remain stable for ca. 1 month when stored in ambient conditions. We have shown that surface-accessible SERS substrates that remain stable for more than 1 year when stored under ambient conditions can be fabricated by physically encapsulating Ag or Au agglomerates in polymer matrices, such as hydroxyethyl cellulose or polycarbophil (Figure 5c).^27−30^ Importantly, these polymers were selected since they do not interact chemically with the nanoparticles and are dissolvable or swellable when treated with solvents, which allows the surface-accessibility of the Ag and Au nanoparticles to be retained for SERS studies. In practice, the encapsulation of the nanoparticles in polymer is simple and only involves mixing powders of the polymer with colloidal nanoparticles to form polymer–nanoparticle liquid suspensions, which could be deposited onto functional materials or dried directly into films to create versatile composite materials useful for SERS studies of real-life targets, such as pesticides, food additives, drugs, and biomolecules.^27−30^

## 6 Surface-Accessible Enhancing Substrates for SERS Studies

**Figure 7:**
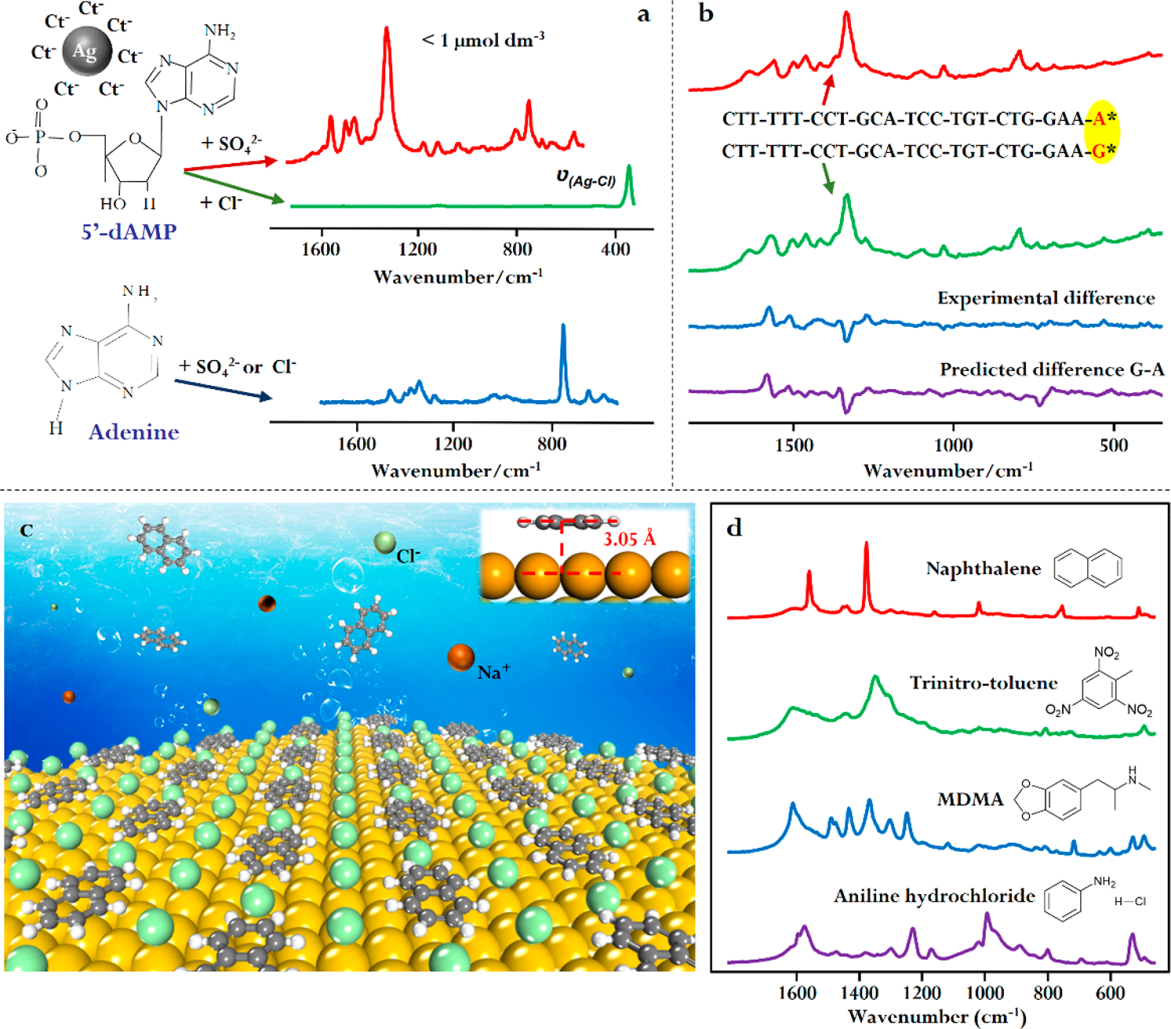
(a) Panel illustrating the effect of strongly bound surface ligands introduced as aggregating agents for the detection of DNA bases and mononucleotides. Adapted with permission from ref (38). Copyright 2006 American Chemical Society. (b) Illustration of the surface-accessible nanoparticle colloids used for SERS difference detection of single base substitution in a 24-mer oligonucleotide. Adapted with permission from ref (37). Copyright 2011 Wiley-VCH Verlag GmbH & Co. KGaA, Weinheim. (c) Schematic diagram of naphthalene coadsorbing with Cl^–^ onto surface-accessible Au nanoparticles assembled at a liquid–liquid interface. Adapted with permission from ref (4). Copyright 2022 Elsevier B. V. (d) Spectra showing the application of π-metal interactions on Au colloids for detection of representative real-life targets. Adapted with permission from ref (31). Copyright 2019 The Royal Society of Chemistry.

In general, surface-accessible Ag and Au nanomaterials provide enhanced functionalities in a wide range of applications,^64,65^ which we illustrate here with examples from our group. An early example was the detection of DNA/RNA and their constituents. The simple nucleobases and nucleosides readily adsorb to citrate-stabilized Ag and Au colloids, but the mononucleotides, which contain both a ribose sugar and a monophosphate group, did not give any signals when the colloids are aggregated using chloride salts. This is due to repulsion between the negatively charged phosphate groups on the nucleotide and the Cl^–^ ions which are more strongly bound to the surface. Conversely, MgSO_4_ gave aggregates that retained weakly bound citrate molecules that could be displaced by the nucleotides. Figure 7a shows that Ag colloids aggregated using NaCl as the aggregating agent gave no signals for 5′-deoxyadenosine monophosphate (5′-dAMP) at concentrations as high as 1000 ppm, but with MgSO_4_ it could be detected at <100 ppm.^38^ This observation was extended to the 5′-deoxynucleotides of all the DNA bases and ultimately to the studies of oligonucleotides and DNA/RNA.^35,39^

Freedom from potential interferences from strongly adsorbed capping ligands is important when the analytes show complex behavior, which depends on their environment or when small differences in spectra are used to uncover subtle changes in DNA/RNA structure. For example, we have found that even simple adenine and adenosine show very rich chemistry on the surface of metal nanoparticles where their spectra can be significantly altered due to the differences in protonation, orientation and even the formation of surface metal complexes that are found at different concentrations and pHs.^32−34^ Similarly, SERS difference spectroscopy can be used to detect signals characteristic of the exchange of a single base in a 24-mer oligonucleotide (Figure 7b).^37^ Indeed, we have recently shown that SERS with colloid can be used to detect guanine oxidation with a sensitivity of 2% from sample droplets as small as 1 μL held on the tip a superhydrophobic wire.^36^

A more recent example that demonstrates the advantage of combining SERS and surface-accessible plasmonic nanomaterials in surface chemistry studies is the discovery of strong π-metal interactions between aryls and group IB metal nanomaterials under ambient conditions.^4,31^ This is important because, despite the ubiquity of aryl molecules in nanotechnology, π-IB metal interactions have previously been believed to be negligible under the ambient conditions where most applications occur.^66^ Using agglomerated citrate-reduced Au colloids as the enhancing substrate, we unexpectedly observed strong SERS signals of a wide range of polyaromatic hydrocarbons, which suggested that these molecules do in fact adsorb spontaneously to Au nanoparticles in solution.^31^ To probe this effect in detail, we carried out further SERS studies using surface-accessible interfacial nanoparticle films as the enhancing substrate.^4^ Importantly, the nanoparticles in the interfacial films were not only unmodified and representative of common Ag and Au colloids but also had access to both the water and oil phases. Using these films, we showed that adsorption was driven by dispersive π-metal interactions rather than hydrophobic forces. Moreover, both the SERS data and density functional calculations showed that the aromatic hydrocarbons did not displace the capping ligands but instead coadsorbed alongside them (Figure 7c). The SERS studies also showed that the π-metal interactions were prevented by covalently adsorbed chemical species such as thiol modifiers. For Ag nanomaterials, the interactions also rapidly diminished in the presence of molecular oxygen due to the formation of a silver oxide shell. These effects account for the lack of interaction observed in the large number of previous SERS studies, where the enhancing substrates were oxidized and/or covered with strongly adsorbed modifiers.^67^ This new understanding paves the way for the rational design of plasmonic sensors with significantly improved sensitivity for important real-life molecules including trinitrotoluene, 3,4-methylenedioxymethamphetamine, and aniline hydrochloride (Figure 7d).

## 7 Conclusions and Outlook

Surface chemistry is at the heart of catalysis, nanotechnology, sensing applications, etc., which continue to attract huge amounts of research activity. While SERS has many potential applications as an analytical technique in real-life applications,^68,69^ the purpose of this Account is to highlight that SERS also possesses many features that make it a powerful technique for surface chemistry studies. Within this context, the mainstream approach for synthesizing SERS enhancing substrates involves using molecular modifiers, which adsorb strongly to the surface of the nanoparticles to direct particle growth and assembly. In studies of molecule–metal interactions, this leads to a conflict between achieving strong enhancement and surface accessibility, which restricts the method to target molecules that can displace modifiers.

In this Account, we discussed our contributions in the development of modifier-free approaches for the synthesis of surface-accessible Ag and Au nanomaterials that enable SERS to be used as a probe for surface chemistry under ambient conditions, although it is important to note that the significance of the surface-accessibility of nanomaterials in applications, particularly in SERS and catalysis, has also been recognized and studied by other research groups, for example, through electrochemical approaches or using the “borrowing strategy”.^48,70^ In our work, we were able to systematically investigate the passivating effect of different modifiers and how the presence of modifiers severely affects the properties and functionalities of the product nanomaterials. Understanding and then overcoming the limitations imposed by the use of modifiers provide new opportunities in manipulating molecule–metal interactions, which can have significant implications in the design and synthesis of next-generation nanomaterials. Most importantly, we hope that the results of our SERS studies will inspire new research in the development of surface-accessible nanomaterials for applications that reach beyond SERS. Indeed, we have shown that many of the modifier-free synthetic approaches discussed in this Account, such as the promoter-induced interfacial self-assembly approach, can be readily applied to applications ranging from catalysis to electronics.
